# Environmental Pollution Effects on Reproductive Health – Clinical-Epidemiological Study in Southern Italy

**Published:** 2012-10-11

**Authors:** M.L. Marra, F. Zullo, B. De Felice, L. Nappi, M. Guida, M. Trifuoggi, C. Nappi, A. Di Spiezio Sardo, B. Zizolfi, G. Capece, F. Visconti, J. Troisi, C. Ciccone, M. Guida

**Affiliations:** 1 Department of Gynecology and Obstetrics, University of Salerno, Salerno, Italy; 2 Department of Life Sciences - Faculty of Mathematical, Physical and Natural Sciences Second University of Naples, Caserta pole, Italy; 3 Department Obstetrics and Gynecology - Faculty of Medicine and Surgery - University of Foggia; 4 Ecotoxicological Observatory - Faculty Biological Sciences - University of Naples Federico II; 5 Department of Chemistry - Faculty of Sciences University of Naples Federico II, Napoli; 6 Department of Gynaecology and Obstetrics, and Pathophysiology of Human Reproduction, University of Naples Federico II, Naples, Italy; 7 Nursing Home Tortorella, Salerno; 8 Laboratory Chemical-merchandising - Special Agency of the Chamber of Commerce of Naples; 9 Department of Obstetrics and Gynecology, St. Joseph Moscati Hospital, Avellino, Italy

## Abstract

This study aims to address the clinical, statistical and Epidemiological Relationship Between Birth Defects and Environmental Pollution, in the Campania Region and in Salerno.

**Objectives:**

We examined four groups of subjects as follows: a sample of pregnant women living in Salerno, a sample of pregnant women living in highly polluted areas, a sample of controls, pregnant women and residents out of the Campania Region, considered in unpolluted areas (Foggia) and in the Salerno area.

**Methodologies:**

a toxicological and genetic analysis was conducted on patients examined.

**Conclusions:**

there is an epidemiological link between environmental pollution and reproductive health in the Salerno area. Experimentally there are the first evidences of endocrine disruptors by the PCB. It has been inferred an overexpression of the mir-191 as a marker of pollution by dioxin-like compounds. Socially, correct information of populations at risk is necessary and a possible preventive and ongoing medical care must be ensured.

## I INTRODUCTION

Identifying the effects of environmental exposures on human health is a major objective of life sciences and biomedical research. In environmental health, the recognition that exposures could produce DNA mutations represented a major landmark for risk assessment and prevention [1]. Consequently, chemical agents have been categorized according to their capability to alter the DNA sequence. Such information has been fundamental to determine environmental risks and shape current regulatory efforts for exposure reduction [2]. Recent evidence suggests that the molecular influence of the environment may extend well beyond the interaction with the DNA sequence [3]. Some studies indicate an increased risk of developing congenital anomalies for fetuses of pregnant women living near waste sites; however, further studies monitoring human levels of xenobiotics, such as PCBs, are needed to demonstrate the relationship with health damage [4]. To date, limited data are available on the Concentrations of PCBs in newborn of women living in municipalities of Salerno with high environmental risk 56 [5]. This is one of the primary direct measure of prenatal exposure to these contaminants [6]. The present study has been aimed to analyze samples of both maternal blood, taken from people resident in towns adjacent to waste site at high environmental impact, in order to determine the levels of PCBs. The towns taken into account in this work were those located in a zone that we call “Pentagon death”, is a large that area includes 39 municipalities of the province of Salerno, which effectively extend the catchment area of Sarno [7].

In the recent years cancer mortality in this area has strongly increased, reaching levels much higher than Italian average [8]. The environmental pollution caused by the discharge of illegal toxic substances from various sources in this region has been hypothesized as a possible cause of increased mortality because high concentrations of PCBs have been often found in the countryside and in the blood of some residents [9]. This is because, since 1980, hazardous waste dumping has been going on for the most part uncontrolled [10]. In Additions to massive amounts of household waste, the region was hit by widespread illegal dumping of toxic industrial chemicals and low-level radioactive waste [11]. While in the river Sarno, since 1993, many large industrial effluents and waste discharges, are released without adequate sewage system [12]. The situation was aggravated by the constant practice of burning waste, which in turn generates dioxins and other toxic compounds [13]. Pregnant women and children living in polluted areas are a high risk group. The risk of exposure to pollutants can be present in uterus (fetal growth period) and the “placental barrier” has proven to be not quite sufficient to prevent chemical compounds [14].

The present study involves a large number of women in the Salerno’s Province carrying out the voluntary termination of pregnancy because of fetal malformations, detected in the second trimester of pregnancy. There are no official data on the total number of all women with malformed fetuses.

The present study will pay particular attention to the serum levels of the “dioxin-like” PCBs, indicated by World Health Organization the likely role of PCBs as toxic that could identify women actually exposed to those pollutants substances in maternal blood [15]. The overall aim of our study has been to verify the possible relationships between pollution and birth defects looking for possible etiopathogenetic mechanisms. So we also took into account medical history, including lifestyle and proximity of highly polluted sites, of the sample populations under study.

In the present study, we evaluated also, the effects of environmental pollution on the expression of mir-191 in maternal blood, measured in healthy women during pregnancy, undergo a therapeutic abortion occurring in the second trimester of pregnancy. Our study aims to Healthy pregnant women find if, without previous illness, but living near landfill sites and exposed to other sources of pollution in the Campania region (Italy), present modification of the expression of Mir-191 compared to an unexposed control group.

## II. METHODOLOGY

### A. Clinical and Epidemiological Analysis

In order to obtain epidemiological data elements two different tracks of data collection have been taken:

1. Questionnaire’s administration dedicated to patients recruited in the study.
2. Data collection of patients subjected to IVG with fetal malformations diagnosis from ‘1 January 2009 to December 31, 2011, in the light of any official publication arrest, about these issues, to December 31, 2008.

#### Questionnaire Administration

The patients recruited for the study were administered a questionnaire, in order to gather all possible information, useful to identify possible associations between exposure and incidence of diseases

#### Survey of epidemiological data

Data relating to age, place of residence, type of malformation, malformations’ presence or absence in family, smoking habits and drugs use, woman’s and her husband’s jobs and ages were collected from some authorized centers to abortions in the second quarter (University Hospital San Giovanni di Dio and Ruggi of Aragon, St. Luke Hospital of Vallo della Lucania, San Giuseppe Moscati Hospital of Avellino),

For the present study were considered aged women between 25 and 35, who held a job in the same place of residence.

For each group belonging to one of the Campania’s Provinces the total malformations’ prevalence has evaluated. For the group from Salerno, has undertaken a further subdivision in order to identify what was the malformations’ prevalence in the considered polluted territory. An attempt to make up the deficiencies of the current epidemiological analyzes of the Salerno area. In fact, the data registry official publication of Campania Region’s congenital anomalies is currently stopped the year 2008. In light of these premises were considered voluntary interruptions of pregnancy, within the limits of the law, received from: the University Hospital St. John of God and of Aragon Ruggi of Salerno the Hospital San Giuseppe Moscati of Avellino St. Luke’s Hospital of Vallo della Lucania

### B. Laboratory Tests

#### Toxicological analysis of maternal blood

Blood samples were examined from 4 groups of patients distributed as follows:

GROUP A: 30 sera taken from pregnant women aged between 20 and 35 years resident in a risk area in Salerno and with fetal malformation
GROUP B: 20 sera taken from pregnant women aged between 20 and 35 years resident in the triangle of death and with fetal malformation diagnosis;
GROUP C1: 10 sera taken from pregnant women aged between 20 and 35 years not living in the Campania Region with healthy fetuses;
C2 GROUP: 10 sera taken from pregnant women in Salerno with healthy fetuses;

#### Maternal blood collection and storing

5 mL fasting samples of venous blood were collected in Vacutainer^®^ tubes from either antecubital vein of mothers by standard phlebotomy techniques. The tubes were centrifuged at 4000 rpm, (centrifuge Mikro 20 Hettich, Germany) for 10 min at 4 ° C and the serum was transferred to a new tube labeled with a identifying code. Glass syringes and glass tubes were used to avoid contamination. The samples were frozen and stored at −20 °C until analyses, to be performed before one week from collection. Control sera were collected from people not resident in Campania Region.

#### Analytical method : standard and reference materials

The choice of congeners to be analyzed was based on 2 principles:

1. The list of 30 congeners (18, 28 +31, 52, 44, 95, 101, 99, 81, 77, 110, 151, 123, 118, 114, 146, 153, 105, 138, 126, 187, 183 +167, 177, 156, 157, 180, 169, 170, 189), established by wHO and below the ISS, based on more PCB products, and therefore more popular, the most persistent, then you probably are still in circulation and the most toxic to humans.
2. The UNI EN 12766:2 2004 which established that the sum of 6 congeners (28, 52.101, 153, 138, 180), multiplied by 5, shows the estimated total concentration of PCBs.

The solvents used, *n*-hexane, methanol, and diethyl ether were of analytical reagent grade and used without any further purification (Sigma-Aldrich, Milan, Italy). The 30 PCBs “dioxin-like” are the congeners identified by the following IUPAC numbers: 18, 28+31, 52, 44, 95, 101, 99, 81, 77, 110, 151, 123, 118, 114, 146, 153, 105, 138, 126, 187, 183+167, 177, 156,157,180,169,170,189. As standards of the 30 congeners we used a mixture in heptane (10 μg/ml of each compound, 97% purity) from Ultrascientific analytical solutions (Bologna, Italy). The internal standards for GC analysis was p,p’DDT (97% purity, 1000 μg/ml in iso-octane) (Ultrascientific analytical solutions (Bologna, Italy).

#### Analytical procedure : Pcb analysis

500.0 μl serum sample, taken back to room temperature, was transferred to a test-tube, added of 5.0 μl of the internal standard (p,p’DDT in iso-octane), and vortexed for a few seconds. One milliliter of methanol was added to the samples and mixed completely by vortex to precipitate the proteins, the extracted with 10 ml of *n*-hexane diethyl ether (1:1 v/v) by rotary mixer (30 min). The supernatant was concentrated to 1 ml under vacuum and purified in SPE Strata FL-PR Florisil (volume: 6ml, 1g packing) and Strata SI-1 Silica (volume: 3 ml, 0.5 g packing) (Phenomenex, Torrance, CA) in serial connection.

It has been washed the column first with 3 ml of distilled water and then with 3 ml of a solution of ammonia at 15%. After drying well the column and passed many ml of air, it was eluted with 3 ml of Iso-Octane and the eluate collected in a new conical tube.

It was brought to dryness with a gentle flow of nitrogen and is prepared to a Florisil column. It is conditioned chromatographic column with 2 ml of toluene.

Once the tube recovery of the whey was brought to dryness with 3 aliquots of 100 l of toluene and it is placed on the silica.

Finally it was necessary to elute with 3 ml of Toluene in a new conical tube.

It is brought again to dryness under a gentle stream of nitrogen and the residue is taken up with two aliquots of 150 l of n-hexane in a conical vial with a capacity of 300 l.

It is capped with a sealing machine and 1 μl injected into the gas chromatographic system.

#### Instrumentation

The analysis of PCBs was performed on a gas chromatograph GC Ultra Trace (ThermoFinningam), equipped with an electron capture detector 63NI (GC-ECD).

The samples were injected by auto sampler equipped with a syringe 10 uL in split mode on a capillary column Zebron ZB-5ms (60m × 0.25mm × 0.25μm thick (Phenomenex Inc., Torrance, CA, USA), using helium gas as carrier gas at a flow rate of 1.1 ml / min, using the following temperature program: 60 ° C initial (1 min) up to 140 ° C to 25 ° C / min and then up to 280 ° C at 2 ° C / min. The detector temperature was set at 300 ° C, while the injector at 280 ° C, the flow of auxiliary gas (nitrogen) was 70 ml / min. Zebron The column was used as main analytical column and the data on the real samples were obtained using this column.

The confirmatory analysis of PCBs was performed on a gas chromatograph spectrometer massaGCMS2010 Plus (Shimadzucorp.), equipped with a detector of single quadruple mass spectrometry.

The samples were injected by auto sampler HTA280T (HTA) equipped with a syringe 10 uL in split mode on a capillary column Zebron ZB-5HT Inferno (30m × 0.1mm × 0.1μm thick (Phenomenex Inc., Torrance, CA, USA), using helium gas as carrier gas at a linear velocity of 50 cm / s, performing the following temperature program: 200 ° C initial (2 min) up to 320 ° C to 20 ° C / min The temperature of the detector was set at 300 ° C, while the injector at 280 ° C, the flow of auxiliary gas (nitrogen) was 70 ml / min. Zebron The column was used as the main analytical column and the data on the samples Real have been obtained using this column.

### C. Genetic analysis

#### RNA Extraction

In samples from individuals resident in Salerno was performed genetic analysis to assess the microRNAs’ expression profiles.

The RNA extraction was performed from maternal blood, taken from the antecubital vein with Vacutainer method, using TRIzol ® Reagent (Life InvitrogenTM by TechnologiesTM), a reagent which allows to isolate total RNA from samples of cells or tissue of human origin, animal, plant, bacterial, or within one hour. It is a monophasic solution of phenol, guanidine isothiocyanate, and other components that facilitate cell lysis allowing RNA extraction while maintaining the integrity, due to inhibition of the activity of ribonuclease while very effective dissolve the cellular components during the ‘homogenisation of the sample. After adding the TRIzol ® Reagent, the samples were incubated at room temperature to allow complete dissociation of the complex core protein.

Thus, it is added chloroform and, after stirring, incubation at room temperature and centrifugation, it was obtained the separation into three phases: an aqueous upper phase (containing the nucleic acids), an interface (containing denatured proteins that are not passed into the lower phase phenolic), and a lower phase pinkish phenol-chloroform (containing cellular debris and proteins). The RNA is present exclusively in the aqueous phase. The upper aqueous phase was transferred to a clean tube and was precipitated by adding isopropanol and the RNA then ethanol. After stirring and centrifugation the supernatant was removed and the pellet was dried and then be resuspended in H2O RNse-free. To determine the yield, concentration, extracted RNA were carried out in absorbance readings at 260nm in a spectrophotometer; the formula A260 × dilution × 40 = μg of RNA / ml allows to obtain the concentration. The index of purity of the extracted RNA is determined by the A260/A280 ratio for a pure RNA should be > 1.8.

#### RT-PCR

The mature miRNAs have been converted into cDNA by reverse transcription. In particular, the method used involves first polyadenylation of the mature miRNAs by the Poly (A) polymerase and then they are reverse-trascripted in c-DNA using primers oligo (dT) that have an anchor sequence at the 3′-terminal and degenerate a universal tag sequence 5′-terminal end. Was prepared a master mix for each sample containing the mixture of nucleotides 10 × (2 μl), the water-free RNase (variable based on the volume of RNA), the buffer with high specificity 5× (4 μl) and reverse transcriptase (2 μl) to which was added to the RNA. The total volume of each reaction was 20 μl). The reverse transcription reaction took place according to a protocol that includes a first thermal incubation at 37 ° C for 60 minutes followed by a second at 95 ° C for 5 minutes, the latter was necessary to inactivate the reverse transcriptase.

#### Real-Time qRT-PCR

The Real Time PCR is the increased development of PCR technology, which is able to detect with reliability and measure in real time the initial concentration of a target sequence in a biological sample. The tools for Real-Time PCR, in addition to act as a thermal cyclers, which are conducted in the amplification reactions, are equipped with a tungsten lamp, which determines the excitation of fluorophores present in the samples, and convey, and then, the fluorescence emitted up to a spectrograph, the reading device at each cycle. Special software acquire the emission spectrum of each sample for the entire duration of the PCR and convert the fluorescence emission in a real time representation of the kinetics of amplification that can analyze the data and convert it into graphs for each reaction. The calculation of the amount of DNA is carried out by determining the PCR cycle (cycle threshold, Ct - threshold cycle -) in which it is reached where the threshold value of fluorescence, i.e., the signals of specific amplification are separable from those of the background noise of the system. The number of cycles necessary for a sample reaches its Ct is inversely proportional to the number of copies of the target initially present. The advantage in terms of precision and range of quantification, compared to conventional PCR, is given by the possibility of quantifying the DNA to the threshold cycle, which is always calculated in the exponential phase of PCR reaction, phase in which the reagents are still far exhaustion and the elements of variability are minimized. With the Real-Time PCR can be carried out a relative quantification by comparing the amount of target copies compared to that of a control gene. The Real-Time PCR can be performed with the use of fluorine hole SYBR Green (C32H37N4S) as a fluorescent molecule that, intercalating into the double-stranded, binds non-specifically to the minor groove of the cDNA; the specificity of the reaction is determined by ‘ analysis of the melting curve (Tm) of the amplicon obtained. The SYBR Green, intercalating into the double helix of DNA which in turn is amplified, emits a fluorescence thousand times greater than that emitted in the absence of DNA. This characteristic makes it ideal for studies of relative quantification of nucleic acids as the fluorescence emitted from the sample that is amplified is directly related to the amount of nucleic acid available initially. The expression of a given transcript should be compared with that of a constitutive gene as this action normalizes the differences that may result from an experimental variability. At the end of amplification, the instrument determines the melting temperature of each amplified product and makes it visible in a graph, in which is shown the intensity of fluorescence as a function of temperature. It is possible, in this way, to highlight the presence of spurious amplification that will present a melting curves different from that of the specific product. In the graph of amplification is given the intensity of fluorescence as a function of the number of cycles and is designated a line that takes the name of Baseline that indicates the value above which begins the accumulation of amplified. This line, in fact, is defined as the cycles in which a fluorescent signal is accumulating but is below the detection limits of the instrument. ΔRn is the increase in the fluorescence signal at each stage. The number of cycles to which each curve intersects the threshold line is indicated as Ct, the cycle of the amplification reaction in which the fluorescence signal of the sample is greater than the background of the bottom. The sample we have the smallest value of the threshold cycle corresponds to that with the amount of cDNA in May. The threshold (threshold) can be set arbitrarily by the operator in a manner as to intersect the curves of all samples in the exponential phase, based on the variability of the Baseline. A signal that is detected above the threshold is considered a true signal that can be used to define the Ct for a sample. The threshold can be adjusted for each experiment so that exponential amplification in the region in all tracks. To prepare the c-DNA for the subsequent quantification of mature miRNAs of interest (miR-191) was carried out the amplification of retroscritti with the primers, of which the reverse “universal” forward and the other for control and for the miRNA of interest. To normalize the amount of miRNA target was used as the endogenous RNA RNU6B, one snRNA has been verified to have relatively stable expression levels in all tissues and cell types. Two mixes were prepared: one containing the primer for the miR-191, and the other with the primer for RNU6B. The reaction was carried out on three dilutions of cDNAs of each sample; each dilution was carried out in duplicate. The reaction mix containing: SYBR Green PCR Master Mix (12.5 μl) × 10 reverse primer (2.5 μl) × 10 forward primer (2.5 μl) and RNase-free water (variable), after the mix can be added to the sample of cDNA (2.5 μl). The plate was loaded and covered with a film. The PCR reaction was conducted using the following protocol (Tab. 3.1):

The phase at 95 ° C is necessary for the activation of DNA polymerase. The phase at 70 ° C improves the collection of the results of fluorescence.

Need to perform the analysis of the dissociation curve of PCR products in order to verify the specificity and identity. The instrument used required the correction of the threshold value obtained to a lower one (0.02) so as to properly analyze the results. The quantification is relative and were calculated by the difference between Ct relative to miR-191 with that on RNU6B.

## III. RESULTS

### A. Results epidemiological analysis

At the time of preparing this study only centers that have the greatest reception from the numerical point of view in the Salerno area are counted (as stated above). Some centers of much smaller impact are still not considered because of the difficulty of access to the respective statistics.

**Fig. 1.**
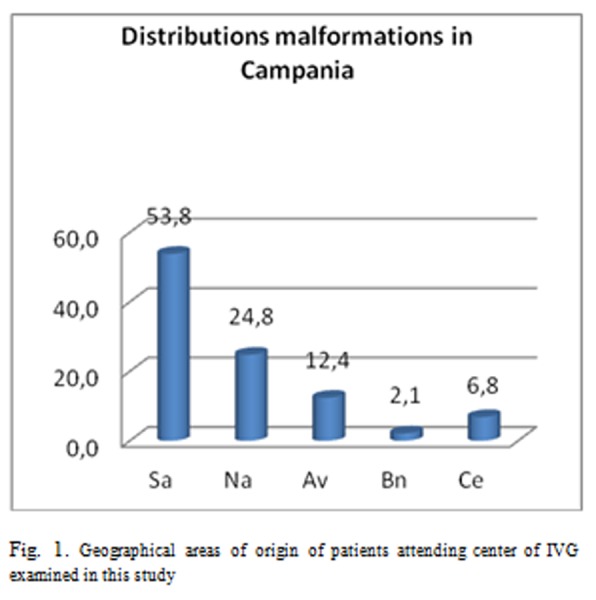
Geographical areas of origin of patients attending center of IVG examined in this study

The analysis of second trimester abortions’ cases recruited in this study has highlighted the data summarized in Fig. 1.

It is evident that of 284 malformations cases performed at the Centers we reviewed, the higher prevalence of 53.8%, was recorded among women living in Salerno’s Province while coming from territories belonging to the Naples’ province was 24.8% and the province of Avellino, 12.4%. We emphasize again that these data are presented for the first time, had not yet been performed, the update of the Birth Defects Registry of Campania Region.

**Fig. 2:**
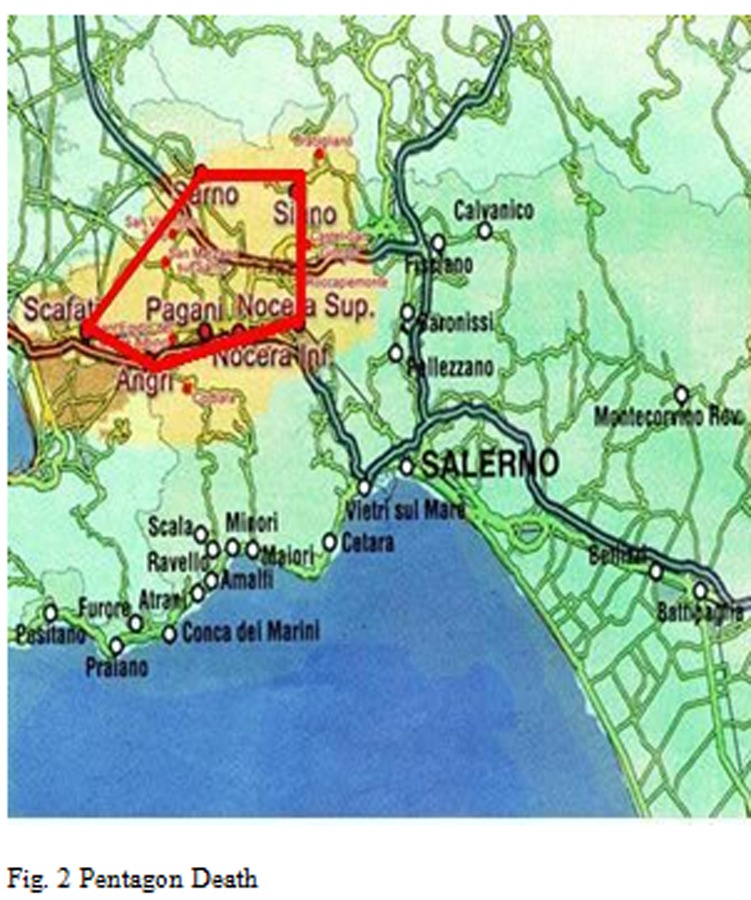
Pentagon Death

**Fig. 3:**
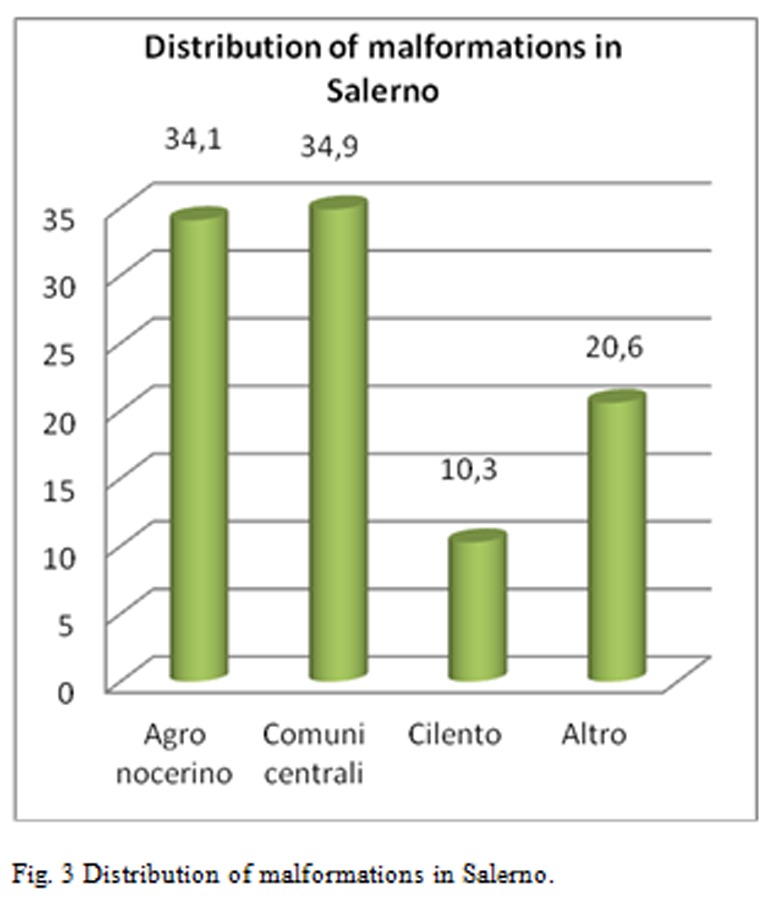
Distribution of malformations in Salerno.

**Fig. 4:**
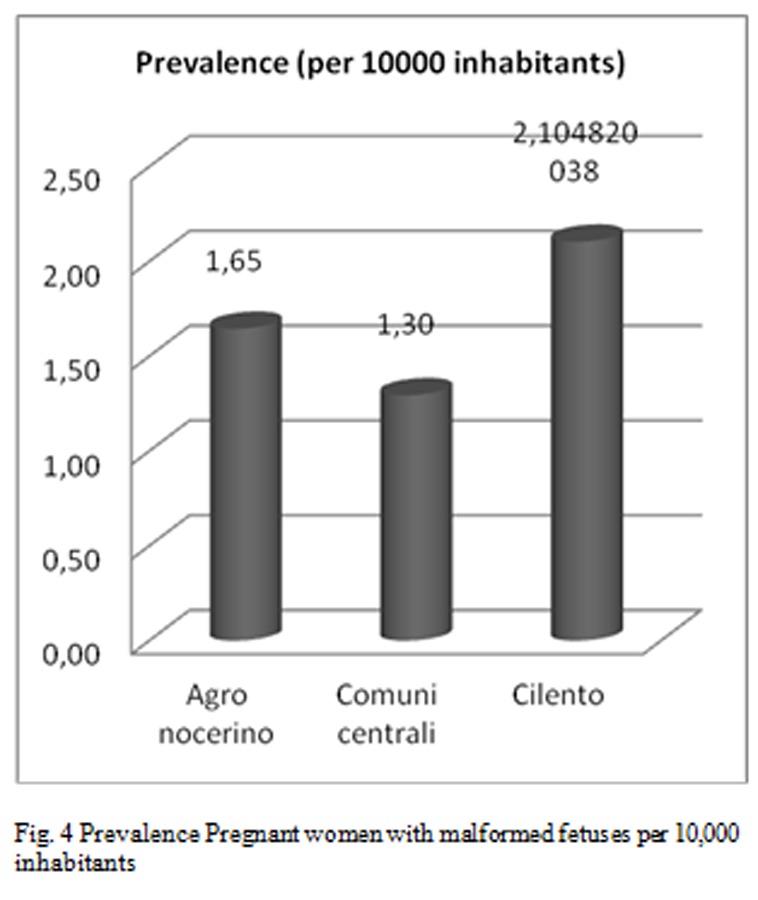
Prevalence Pregnant women with malformed fetuses per 10,000 inhabitants

**Fig. 5:**
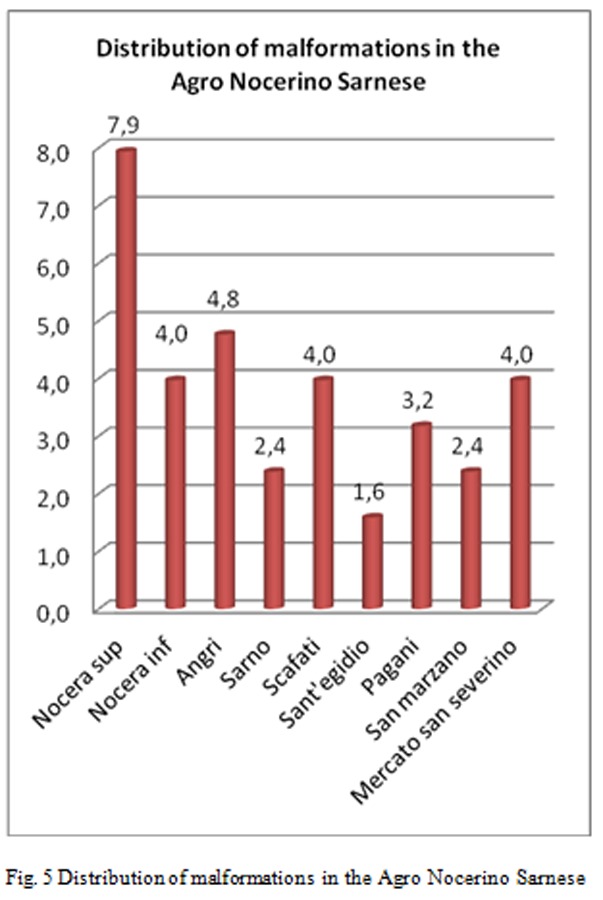
Distribution of malformations in the Agro Nocerino Sarnes

**Fig. 6:**
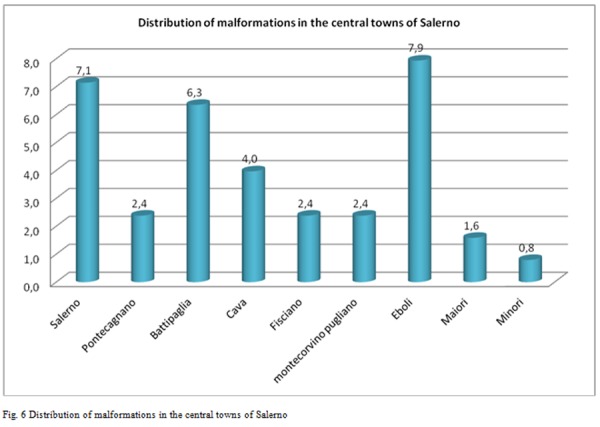
Distribution of malformations in the central towns of Salerno

Breaking down the analysis further, and going to investigate the Salerno’s Province, patients origin’s territories who flocked to these centers for IVG are identified as follows Fig. 1, Fig. 2, Fig. 3, Fig. 4, Fig. 5, Fig. 6.

The processed data correlate well with the known Salerno’ municipalities’ high pollution rate, but not specifically reported in the literature, supporting the hypothesis of an increased prevalence of malformations in these highly polluted sites.

### B. Laboratory Analysis Results

#### Results of toxicological

**Fig. 7:**
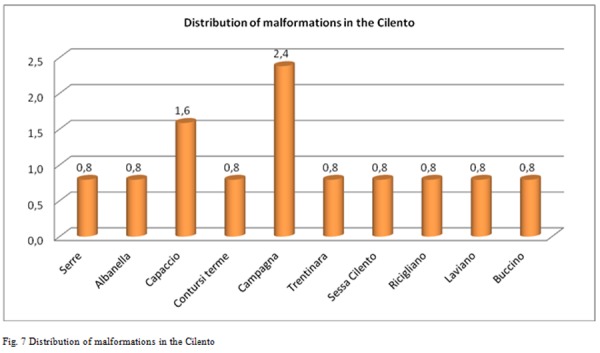
Distribution of malformations in the Cilento

The Fig. 7 shows the graphical representation of the PCB congeners presence in patients, respectively, attributable to the Triangle of death, Salerno and controls.

**Fig. 8:**
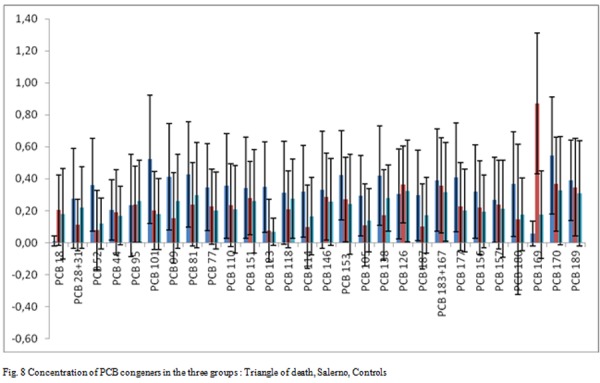
Concentration of PCB congeners in the three groups : Triangle of death, Salerno, Controls

The Fig. 8 shows the estimated total of PCB in the three groups

**Fig. 9:**
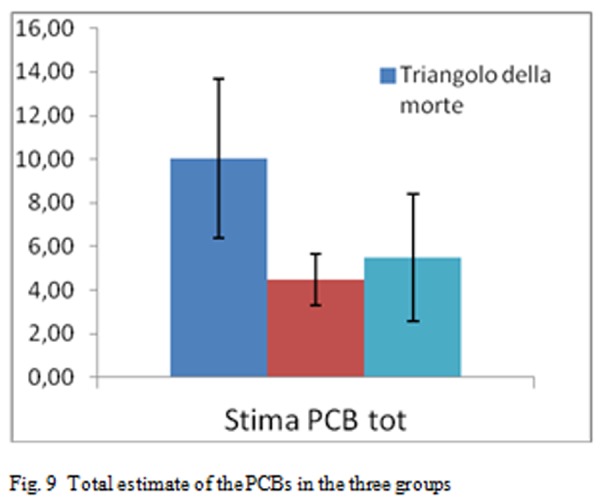
Total estimate of the PCBs in the three groups

The Fig. 9 shows the mean concentration of the PCB 169 in the three groups

The shows the PCB analyzed, the angular coefficients (a), the values of the intercept (b), the value of the correlation coefficient (R2) and the number of observation (n), the recovery %, the LOD (limit of detection ) and LOQ (limit of quantification).

All the case studied have a R2 value at least 0.999.

The LOD varies slightly between the PCB and has been calculated to be on average <0.05 while the LOQ was generally less than 0.1 ng / ml.

The average yield of the method was found to be 95.1% with a minimum value of 85% and a maximum value of 106%.

The intra-day, carried out on five sera, with all congeners, showed a variation coefficient of 1% on average (Pcb: 18, 28 +31, 52, 44, 95, 101, 99, 81, 77, 110, 151, 123, 118, 114, 146, 153, 105, 138, 126, 187, 183 +167, 177.156, 157, 180, 169, 170, 189, while the inter-day reported an average coefficient of variation 3% (PCB: 18, 28 +31, 52, 44, 95, 101, 99, 81, 77, 110, 151, 123, 118, 114, 146, 153, 105, 138, 126, 187, 183 + 167, 177, 156, 157, 180, 169, 170, 189), it served to establish that the method was reproducible.

The 30 different congeners were examined in 3 groups. The sera’s number percentage with detectable level of each PCB congener, the average value, the minimum value and the maximum value, the standard deviation for the PCB seen in more than one serum analyzed and the total content of PCB (sum of the 6 congeners multiplied by 5) are shown for the 3 groups.

For Group A (patients living in the province of Salerno), the presence of PCBs were found in all test sera; The congener 169 is found in quantities higher average 0.69 g / ml. The 169 congener is much higher in this group compared to the other two (as confirmed by the z-test). The total content of PCBs is 4.50 ng / ml, less than the first group and approximately equal to control.

For Group B (patients residents in the risk area), the PCBs’ presence was found in all sera examined. The average content of total PCBs was found to be 10.03 ng / ml, the highest among all groups.

Then in the province of Naples the total amount of PCB is greater than the other groups (as confirmed with the z-test). Has not been registered any PCB congener more frequent as each type of congeners has been detected in all sera.

For the third group, the presence of PCBs were found in all test sera, did not register any PCB congener most frequently as each type of congeners was detected in all sera. The total content of PCBs is 5.47 ng / ml, less than the first group and approximately equal to the second (as confirmed by the z-test).

**Fig. 10:**
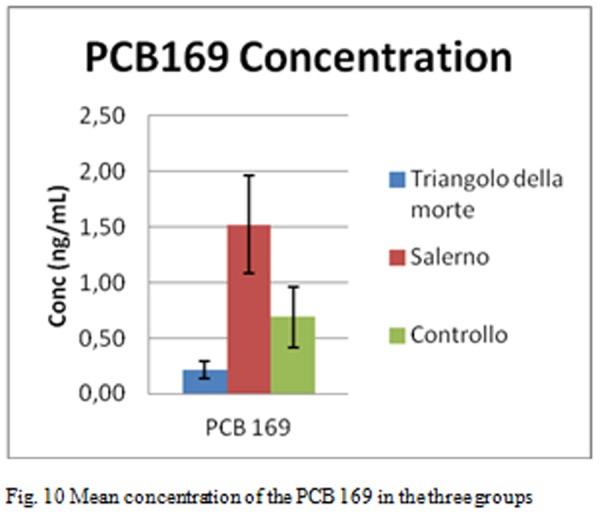
Mean concentration of the PCB 169 in the three groups

**Fig. 11:**
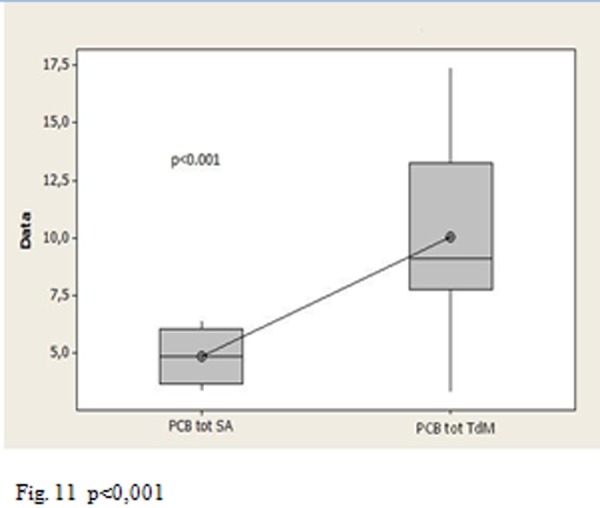
p<0,001

**Fig. 12:**
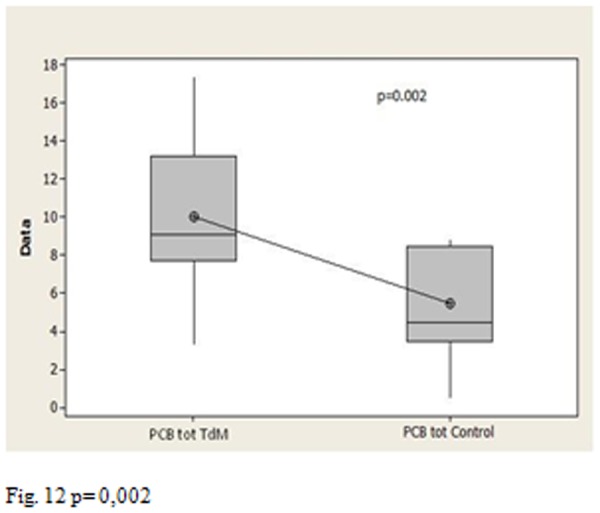
p= 0,002

The p-values allow us to affirm that the total PCB concentration was statistically higher than in Triangle of death to Salerno (Fig. 10 p <0.001), there is a statistically significant difference between the Triangle of death’s group and the control group (Fig. 11 p = 0.002), no statistically significant difference between the group of Salerno and the group of controls (Fig. 12 p = 0.05).

**Fig. 13:**
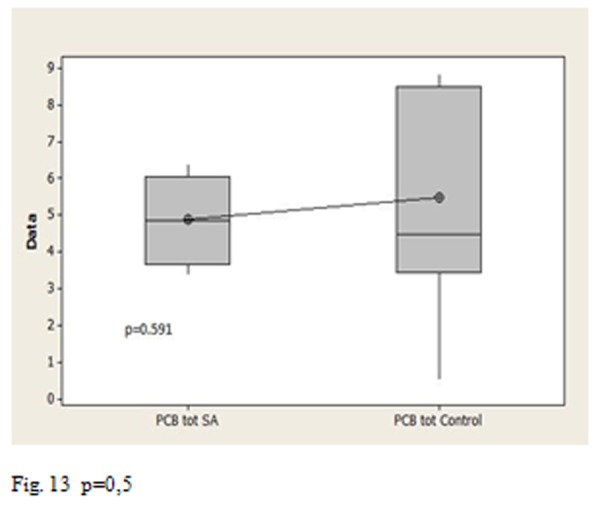
p=0,5

**Fig. 14:**
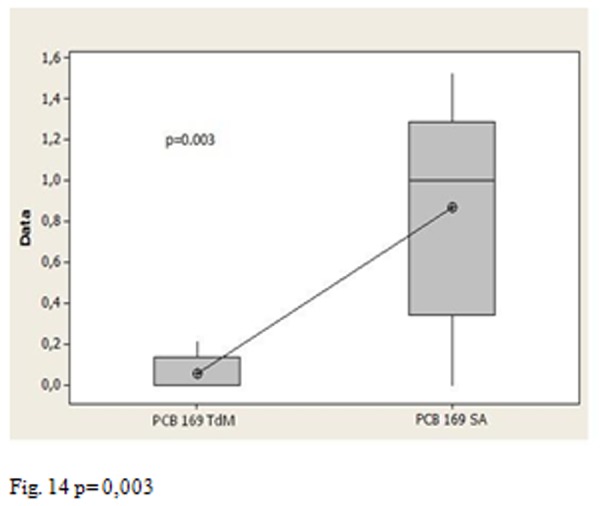
p= 0,003

Moreover the p values allow us to affirm that the average amount of congener 169 in Salerno group is statistically higher than the Triangle of death’s group (Fig. 13 P = 0.003), the group of Salerno has an average content of congener 169 also statistically higher compared to control (Fig. 14 p = 0.008), there is a statistically significant difference between the average content of congener 169 in the Triangle of death’s group and the control groups (Fig. 15 p = 0.234 17)

### 3.3 Genetic analysis results

#### 3.3.1 Genetic analysis results

Following the results of PCBs’ content in the patient group of women living in the Salerno with malformed fetuses, we have deepened the study with the analysis of the profiles of miRNA in this group and in the control, in order to compare the expression of Mir-191.

Mir-191 for all samples tested, with high concentrations of 169 congener, shows that overexpression was to be an average of 4.1 times compared to the expression in the control group.

The expression of the Mir-191 in the Salerno patients compared to controls is shown in **Fig. 16**

The sensitivity of the technique has made it possible to detect even minimal concentrations of Mir-191 and to affirm that its over-expression is statistically significant compared to the control group.

The results obtained allow to state that the Mir-191 is closely correlated with exposure to dioxin and dioxin substances - like (PCB 169).

Although the survey conducted on maternal blood lab only reported high concentrations of PCB congener 169 for, through the results of Real Time PCR has been confirmed so far reported only in few studies in literature, that the Mir-191 is up-regulated following exposure to concentrations of pollutants, particularly dioxin-like PCBs.

The data evinced could make us assume the possibility that the Mir-191 represents a marker of pollution, and more precisely, of exposure to dioxin.

It takes into account, however, the limited numerical strength of these groups in evaluating the results, since they represent only part of the results of a preliminary study of a larger project is not finished yet.

## IV DISCUSSION

The obtained data from the various arms of this study deserves consideration and caution. In fact, in order to obtain valid conclusions in both the scientific perspective and in social and epidemiological ones the current results need to be framed in a broader context. Today access to sources is not available in fact the present literature is not yet able to provide the necessary support. The scientific community is, therefore, still agree about the impossibility of identifying evidence that link the environmental pollution impact on human reproduction.

However, the assumptions made on the basis of this study, formulated and supported by scientific evidence, allow to the possibility of reaching important experimental evidence, several other ideas for consideration, but first of all a socio - political and - statistical epidemiology.

A. First, about the toxicological analysis of maternal blood should be emphasized that the serum levels PCbs’ assessment allowed in the present study, to obtain the necessary premises for the genetic evaluation, although it is a complex procedure that requires appropriate methods.

We examined 30 dioxin-like congeners, commonly found in industrialized countries according to WHO. We found high average concentration of congener 169 in Salerno, which suggests that industrial activity, environmental contamination, illegal practices to control waste disposal, although not well manifest according to the literature, an unmistakable mark have left. It is also conceivable that the congener 169, already considered in the literature as the most toxic of the dioxin derivatives, can be used as a marker of environmental pollution.

B. One of the most encouraging aspects from this study is about the miRnas’ expression in serum of malformed fetuses’ mothers in Salerno’ province, residents in areas most exposed to environmental pollution indicated by high levels of PCBs.

As is apparent from the statistical analysis reported in the previous paragraphs, there is no doubt that the Mir-191’s overexpression is accompanied by the presence of fetal malformations in pregnancies that have arisen in the territories polluted Salerno.

This does not occur in mothers of healthy fetuses.

This study deserves to be deepened, especially in perspective below:

1. Increase the number of cases studied in both areas at risk as well as outside of them
2. Mirna’s specific attribution to specific individual fetal malformations
3. Rigorous comparison between the congeners of PCBs’s levels and selected Mirna’s levels
4. Continuous monitoring of the international literature about the actual clinical significance of changes in expression profiles of Mirna.

It is clear that if it is further confirmed the ‘hypothesis of correlation between environmental pollution, overexpression of mir-191 and fetal malformations, a preventive intervention, a socio-political and administrative protection of the resident childbearing age population in areas at risk must be applied.

C. Furthermore, it is important to report that statistical-epidemiological analysis highlighted important data: in the Salerno area, a large proportion of malformed fetuses are from Agro Nocerino-Sarnese. That makes it evident that the environmental and health conditions of the entire area on the valley of the River Sarno persist to be associated with effects on human health and in particular show a significant correlation with the occurrence of fetal malformations. On the other hand, must again be stressed that the environmental situation of the territory about the “pentagono of death” was subject of numerous media reports that have highlighted the precariousness of the environment and that can not be underestimated.

D. Finally, about the socio-political point of view, this study has allowed to recognize those who are the weaknesses of social and health planning in the management of issues about the impact of environmental pollution on human health in Campania and in particular in province of Salerno. So we highlighted a critical collection data at regional and provincial level, both about sources of exposure to toxins and pollutants, both about a strict classification of adverse events and their possible links with the environment.

It follows from these considerations, the strong appeal to policymakers, an implementation for health care and for epidemiological surveys, although the well-known shortcomings of economic and human resources, could be a way to save money, where it were possible to eliminate the causes of disease rather than simply treat them.

Our study therefore allows to invoke the following initiatives:

1. Precise location of the areas actually exposed to environmental pollutants and never been investigated by methodological rigor
2. Development of detectors epidemiological on health of the citizens residing
3. Strict monitoring of pollutant effects about exposure, both for their births, both for their unborn
4. Rigorous nosographic control of malformations on embryos and fetuses, whether born or unborn.
5. Rigorous classification of the individual types of malformations
6. Restructuring of the statistical tests about fetal malformations.

## V CONCLUSIONS

It may be assumed that the present study is a pilot-project study about the reproductive health consequences of environmental pollution, although it is still impossible to reach an unambiguous interpretation from data obtained by the various arms of this study. They, however, offer the possibility to implement larger projects and extensive efforts to face all the concomitant effects about short, medium and long term effects to infants apparently healthy, about adults’ possible interferences, which are represented by genetic constitution (with possible weaknesses or predispositions), the details of life (related to concomitant harmful effect of drug addiction, alcohol abuse, the poor eating habits and exercise) and finally by their work (which is often not well controlled in profile of protection and safeguard her health workers), in addition to residence.

The contribution of scientific research will not be irrelevant to the actual conditions of life and health of the population living in areas at environmental risk

## Figures and Tables

**Fig. 15:**
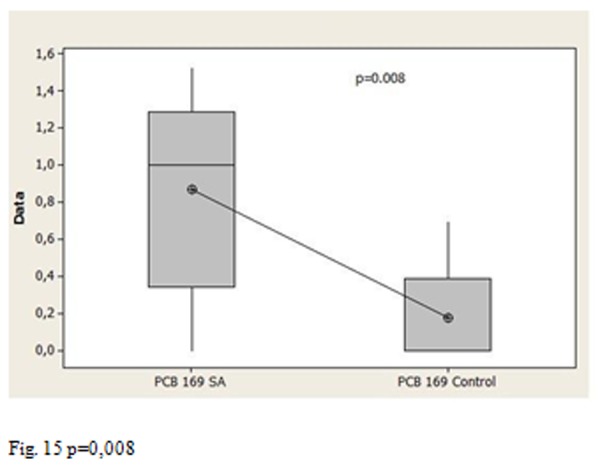
p=0,008

**Fig. 15:**
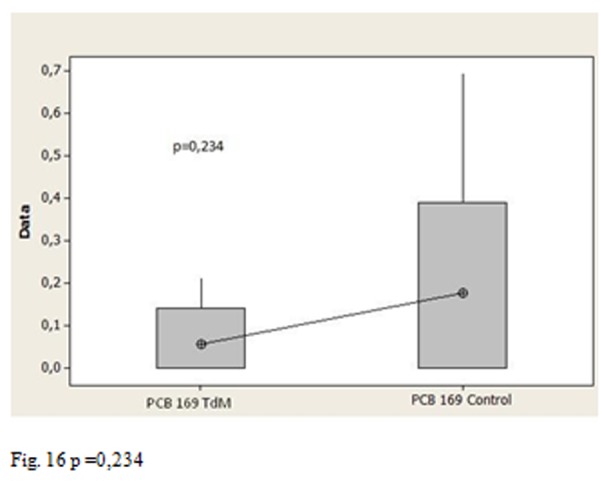
p =0,234

**Fig. 17:**
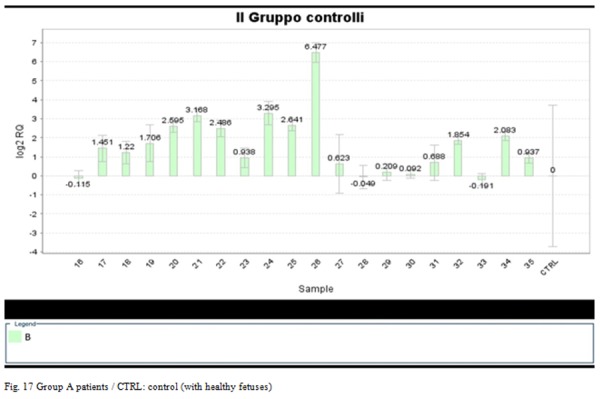
Group A patients / CTRL: control (with healthy fetuses)

**Tab. 1:** PARAMETERS OF EVALUATION FOR EACH PCB CONGENER

**Analyte**	**a**	**b**	**R^2^**	**Recovery (%)**	**LOD (ng/mL)**	**LOQ (ng/mL)**
**PCB 18**	525.4	30.1	0.99989	86	0.05	0.1
**PCB 28 + PCB 31**	1352.5	−54.3	0.99912	88	0.05	0.1
**PCB 52**	956.4	12.5	0.99945	91	0.05	0.1
**PCB 44**	886.2	32.6	0.99977	95	0.05	0.1
**PCB 95**	1012.3	41.7	0.99920	94	0.05	0.1
**PCB 101**	1164.5	−21.6	0.99999	95	0.05	0.1
**PCB 99**	985.3	−41.7	0.99931	95	0.02	0.1
**PCB 81**	976.4	31.8	0.99971	97	0.05	0.1
**PCB 77**	854.3	38.3	0.99963	102	0.05	0.1
**PCB 110**	798.6	47.7	0.99994	106	0.02	0.1
**PCB 151**	996.4	−46.4	0.99985	101	0.02	0.1
**PCB 123**	324.3	−40.8	0.99988	94	0.02	0.1
**PCB 118**	949.6	41.6	0.99979	96	0.05	0.1
**PCB 114**	913.4	47.1	0.99984	96	0.05	0.1
**PCB 146**	992.5	44.7	0.99925	97	0.05	0.1
**PCB 153**	820.5	32.0	0.99923	89	0.05	0.1
**PCB 105**	885.0	−37.6	0.99966	88	0.05	0.1
**PCB 138**	902.0	35.1	0.99969	103	0.02	0.1
**PCB 126**	905.1	38.0	0.99920	87	0.02	0.1
**PCB 187**	931.0	−37.2	0.99975	105	0.02	0.1
**PCB 183 + PCB 167**	930.6	33.0	0.99992	100	0.05	0.1
**PCB 177**	727.9	35.0	0.99907	99	0.05	0.1
**PCB 156**	819.9	−44.4	0.99905	98	0.05	0.1
**PCB 157**	876.1	47.0	0.99964	85	0.05	0.1
**PCB 180**	921.4	−39.8	0.99992	92	0.05	0.1
**PCB 169**	729.2	−35.3	0.99943	93	0.05	0.1
**PCB 170**	859.1	38.2	0.99929	91	0.05	0.1
**PCB 189**	888.6	53.4	0.99983	101	0.05	0.1

**Tab. 2:** DISTRIBUTION OF PCB CONGENERS IN GROUP A PATIENTS

**PCB Congener**	**Percentage of each PCB in the total sampled**	**Average ng/ml (μ)**	**Standard Deviation (σ)**	**Min-Max ng/ml**
PCB 18	8,33	0,14	0,22	0,00 – 0,64
PCB 28+31	0,00	0,07	0,16	0,00 – 0,62
PCB 52	8,33	0,05	0,25	0,00 – 0,44
PCB 44	66.67	0,32	0,27	0,00 – 0,77
PCB 95	41,67	0,24	0,24	0,00 – 0,68
PCB 101	58,33	0,20	0,24	0,00 – 0,54
PCB 99	50,00	0,27	0,28	0,00 – 0,76
PCB 81	41,67	0,30	0,26	0,00 – 0,77
PCB 77	58,33	0,25	0,24	0,00 – 0,63
PCB 110	41,67	0,17	0,26	0,00 – 0,79
PCB 151	58,33	0,25	0,23	0,00 – 0,80
PCB 123	50,00	0,17	0,20	0,00 – 0,53
PCB 118	50,00	0,23	0,24	0,00 – 0,58
PCB 114	50,00	0,24	0,26	0,00 – 0,72
PCB 146	66,67	0,30	0,27	0,00 – 0,72
PCB 153	50,00	0,26	0,26	0,00 – 0,76
PCB 105	16,67	0,13	0,26	0,00 – 0,63
PCB 138	50,00	0,27	0,28	0,00 – 0,72
PCB 126	33,33	0,24	0,24	0,00 – 0,66
PCB 187	50,00	0,13	0,27	0,00 – 0,37
PCB 183+167	66,67	0,39	0,30	0,00 – 0,78
PCB 177	41,67	0,25	0,28	0,00 – 0,72
PCB 156	58,33	0,34	0,29	0,00 – 0,78
PCB 157	41,67	0,25	0,28	0,00 – 0,80
PCB 180	41,67	0,18	0,47	0,00 – 0,65
PCB 169	75,00	0,69	0,44	0,00 – 1,52
PCB 170	66,67	0,37	0,29	0,00 – 0,75
PCB 189	58,33	0,36	2,46	0,00 – 0,80
**Estimated total PCB**	**4,50 ng/ml**			

**Tab. 3:**
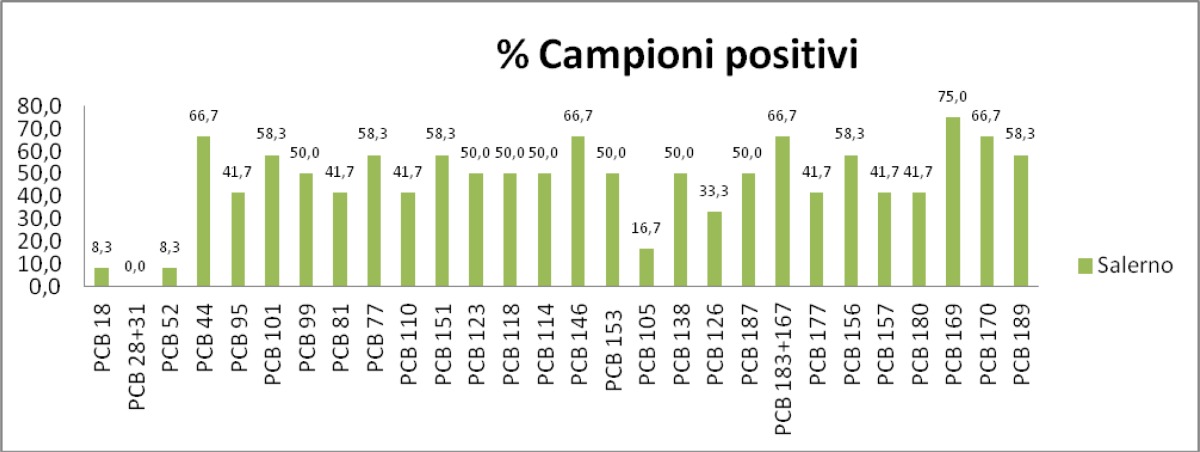
PERCENTAGE DISTRIBUTION OF EACH CONGENER IN GROUP A

**Tab. 4:** DISTRIBUTION OF PCB CONGENERS IN GROUP B PATIENTS.

**PCB Congener**	**Percentage of each PCB in the total sampled**	**Average ng/ml (μ)**	**Standard Deviation (**σ**)**	**Min-Max ng/ml**
PCB 18	10,53	0,01	0,03	0,00–0,10
PCB 28+31	57,89	0,28	0,31	0,00–0,82
PCB 52	84,21	0,36	0,29	0,00–0,89
PCB 44	63,16	0,20	0,19	0,00–0,52
PCB 95	47,37	0,23	0,32	0,00 – 0,90
PCB 101	89,47	0,52	0,40	0,00 – 1,52
PCB 99	68,42	0,41	0,33	0,00 – 0,78
PCB 81	84,21	0,43	0,33	0,00 – 0,87
PCB 77	73,68	0,35	0,27	0,00 – 0,77
PCB 110	68,42	0,36	0,33	0,00 – 0,88
PCB 151	63,16	0,34	0,32	0,00 – 0,88
PCB 123	73,68	0,35	0,28	0,00 – 0,84
PCB 118	68,42	0,31	0,32	0,00 – 0,86
PCB 114	73,68	0,32	0,29	0,00 – 0,82
PCB 146	52,63	0,33	0,37	0,00 – 0,85
PCB 153	78,95	0,42	0,28	0,00 – 0,90
PCB 105	73,68	0,29	0,25	0,00 – 0,69
PCB 138	73,68	0,42	0,31	0,00 – 0,89
PCB 126	63,16	0,30	0,28	0,00 – 0,88
PCB 187	73,68	0,30	0,28	0,00 – 0,86
PCB 183+167	84,21	0,39	0,32	0,00 – 0,85
PCB 177	78,95	0,41	0,34	0,00 – 0,90
PCB 156	63,16	0,32	0,29	0,00 – 0,81
PCB 157	73,68	0,27	0,27	0,00 – 0,85
PCB 180	68,42	0,37	0,33	0,00 – 1.12
PCB 169	36,84	0,06	0,08	0,00–0,21
PCB 170	89,47	0,55	0,37	0,00 – 0,99
PCB 189	84,21	0,39	0,25	0,00 – 0,77
**Estimated total PCB**	**10,03 ng/ml**			

**Tab. 5:**
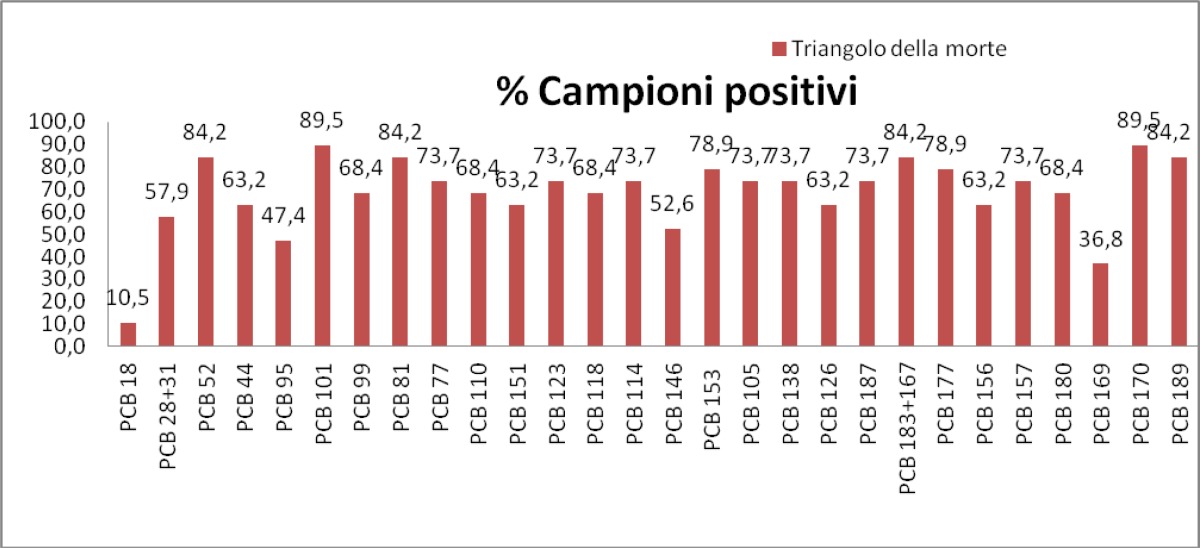
PERCENTAGE DISTRIBUTION OF EACH CONGENER IN GROUP B

**Tab. 6:** DISTRIBUTION OF PCB CONGENERS IN PATIENTS OF GROUP C

**PCB Congener**	**Percentage of each PCB in the total sampled**	**Average ng/ml (μ)**	**Standard Deviation (σ)**	**Min-Max ng/ml**
PCB 18	33,33	0,18	0,28	0,00 – 0,64
PCB 28+31	55,56	0,22	0,25	0,00 – 0,62
PCB 52	44,44	0,12	0,16	0,00 – 0.44
PCB 44	55,56	0,17	0,18	0,00 – 0,45
PCB 95	55,56	0,26	0,26	0,00 – 0,62
PCB 101	55,56	0,18	0,22	0,00 – 0,54
PCB 99	55,56	0,26	0,29	0,00 – 0,76
PCB 81	55,56	0,30	0,33	0,00 – 0,77
PCB 77	55,56	0,20	0,24	0,00 – 0,63
PCB 110	55,56	0,21	0,27	0,00 – 0,79
PCB 151	55,56	0,26	0,32	0,00 – 0,80
PCB 123	44,44	0,07	0,09	0,00 – 0,21
PCB 118	66,67	0,28	0,25	0,00 – 0,60
PCB 114	44,44	0,17	0,24	0,00 – 0,71
PCB 146	55,56	0,26	0,27	0,00 – 0,68
PCB 153	44,44	0,24	0,31	0,00 – 0,76
PCB 105	44,44	0,14	0,20	0,00 – 0,46
PCB 138	77,78	0,28	0,21	0,00 – 0,60
PCB 126	55,56	0,32	0,32	0,00 – 0,66
PCB 187	55,56	0,17	0,24	0,00 – 0,73
PCB 183+167	66,67	0,32	0,31	0,00 – 0,78
PCB 177	44,44	0,20	0,26	0,00 – 0,65
PCB 156	55,56	0,20	0,23	0,00 – 0,64
PCB 157	44,44	0,21	0,30	0,00 – 0,76
PCB 180		0,18	0,23	0,00 – 0,65
PCB 169	44,44	0,18	0,27	0,00 – 0,69
PCB 170	55,56	0,33	0,34	0,00 – 0,75
PCB 189	55,56	0,31	0,33	0,00 – 0,80
**Estimated total PCB**	**5,47 ng/ml**			

**Tab. 7:**
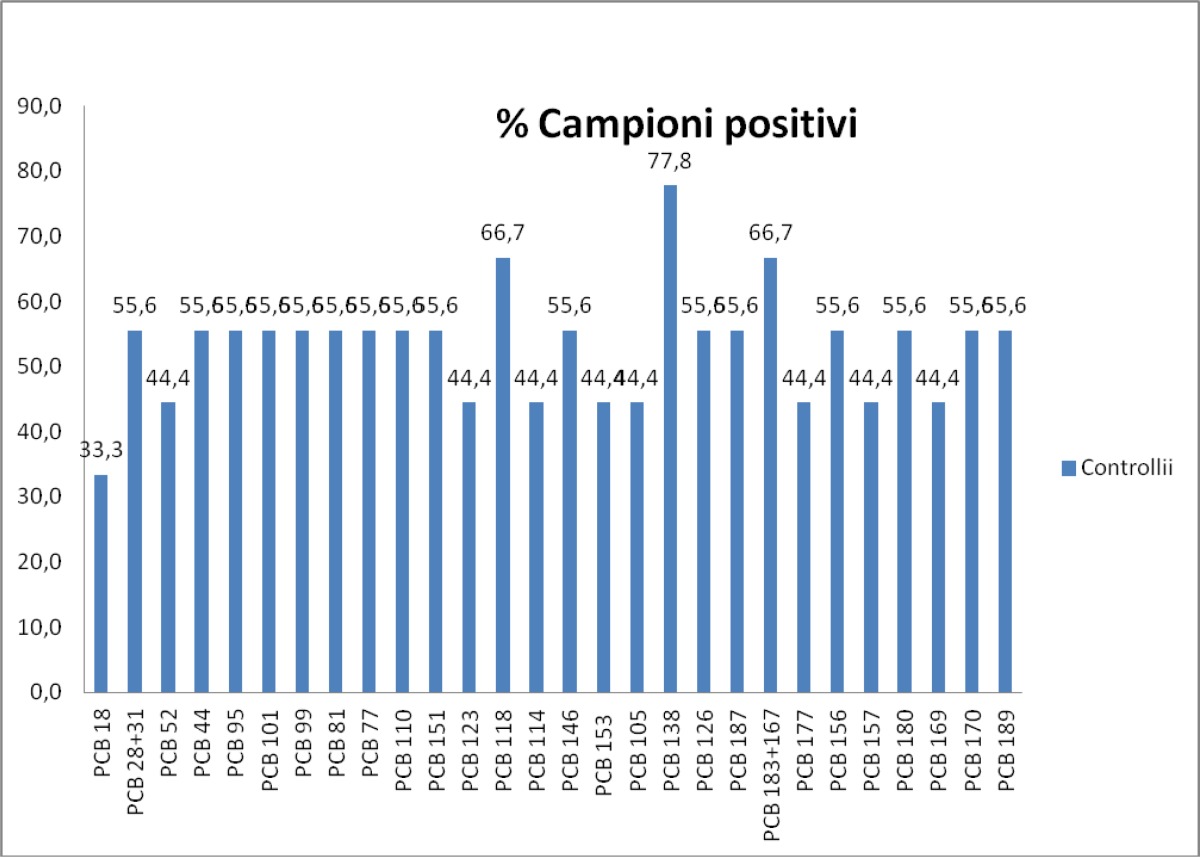
PERCENTAGE DISTRIBUTION OF EACH CONGENER IN GROUP C
